# P-1806. Incidence of BK Virus Nephropathy in Kidney Transplant Recipients

**DOI:** 10.1093/ofid/ofaf695.1975

**Published:** 2026-01-11

**Authors:** Julia Ramponi, Fabián Herrera, Gustavo A Castro Torres, Maximiliano Gabriel Castro, Pablo Bonvehí, Diego Torres, Agustina Fiori, Nicolás Lasserre, Marcia Querci, Elena Temporiti

**Affiliations:** CEMIC: Centro de Educacion Medica e Investigaciones Clinicas Norberto Quirno, Ciudad autónoma de Buenos Aireis, Ciudad Autonoma de Buenos Aires, Argentina; Centro de Educación Médica e Investigaciones Clínicas, CEMIC, BUENOS AIRES, Ciudad Autonoma de Buenos Aires, Argentina; CEMIC: Centro de Educacion Medica e Investigaciones Clinicas Norberto Quirno, Ciudad autónoma de Buenos Aireis, Ciudad Autonoma de Buenos Aires, Argentina; CEMIC: Centro de Educacion Medica e Investigaciones Clinicas Norberto Quirno, Ciudad autónoma de Buenos Aireis, Ciudad Autonoma de Buenos Aires, Argentina; CEMIC: Centro de Educacion Medica e Investigaciones Clinicas Norberto Quirno, Ciudad autónoma de Buenos Aireis, Ciudad Autonoma de Buenos Aires, Argentina; Centro de Educación Médica e Investigaciones Clínicas, CEMIC, BUENOS AIRES, Ciudad Autonoma de Buenos Aires, Argentina; CEMIC: Centro de Educacion Medica e Investigaciones Clinicas Norberto Quirno, Ciudad autónoma de Buenos Aireis, Ciudad Autonoma de Buenos Aires, Argentina; CEMIC: Centro de Educacion Medica e Investigaciones Clinicas Norberto Quirno, Ciudad autónoma de Buenos Aireis, Ciudad Autonoma de Buenos Aires, Argentina; Hospital Universitario CEMIC, Buenos Aires, Ciudad Autonoma de Buenos Aires, Argentina; Hospital Universitario CEMIC, Buenos Aires, Ciudad Autonoma de Buenos Aires, Argentina

## Abstract

**Background:**

BK virus (BKV) reactivation in urine and plasma occurs in 30–60% and 4.5–27% of kidney transplant (KT) recipients, respectively. Of these, 1–5% will develop BKV-associated nephropathy (BKVAN), which can lead to graft loss. BKVAN is defined as histological changes in the graft secondary to BKV reactivation.

**Methods:**

This was a prospective cohort study. KT recipients with BKV reactivation in urine and plasma confirmed by quantitative PCR at a University Hospital (January 2020 – December 2024) were included. Chi-square tests were used for categorical variables and Student’s t-test for differences in means and medians; interquartile range (IQR) was reported for continuous variables.

**Results:**

During the study period, 202 KTs were performed, of which 68 (33.7%) had BKV reactivation in urine, and 27 (13.4%) also in plasma. BKV reactivation occurred more than once in 34.1% of cases. Sixteen recipients (23.5%) developed BKVAN. Of these, 81.3% were male. The median age was 53.9 years (IQR 50.5–57.2). Donor type was deceased (75%), unrelated living (5.9%), and related living (19.1%). Induction immunosuppression included corticosteroids (100%), thymoglobulin (86%), mycophenolate (82.8%), tacrolimus (10.9%), and rituximab (4.7%). 6.8% of KT receptors had concurrent CMV infection. Among BKVAN patients, 81.3% experienced rejection and 14.3% lost the graft. The median time from transplant to BKVAN diagnosis was 143 days (IQR 83–853). Recent organ rejection was the only factor associated with BKVAN (p< 0.0001). Clinical presentation included renal insufficiency (56.8%), dysuria (15.9%), hematuria (4.5%), and fever (2.3%). Treatment involved reduction of immunosuppression (77%), regimen changes (40.9%), and leflunomide administration (37%). No hospitalizations or BKVAN-related deaths were recorded.

**Conclusion:**

The incidence of BKVAN was higher than that reported in the literature. Rejection was the only factor associated with BKVAN, suggesting its role in disease progression.

**Disclosures:**

All Authors: No reported disclosures

